# Effect of Different Medications on Capsule Formation Around Miniaturized Breast Implants in Murine Models–a Systematic Review

**DOI:** 10.1007/s00266-025-04742-x

**Published:** 2025-03-06

**Authors:** Theodor Mareş, Guido Firmani, Cristian Radu Jecan, Fabio Santanelli di Pompeo, Michail Sorotos

**Affiliations:** 1 Department of Plastic and Reconstructive Surgery, Sant’Andrea Hospital, NESMOS (Neurosciences, Mental Health and Sensory Organs) Department, Faculty of Medicine and Psychology, Azienda Ospedaliera Sant’Andrea – U.O.D. Chirurgia Plastica, Sapienza University of Rome, Via di Grottarossa 1035/1039, 00189 Rome, Italy; 2 Carol Davila University of Medicine and Pharmacy, Bucharest, Romania; 3 Department of Plastic and Reconstructive Surgery, “Prof. Dr. Agrippa Ionescu” Clinical Emergency Hospital, Bucharest, Romania; 4 Sapienza University of Rome, Rome, Italy

**Keywords:** Breast implants, Capsular contracture, Gel bleeding, Pharmacological interventions

## Abstract

**Background:**

Breast implants (BIs) lead to the formation of a periprosthetic capsule, causing complications like capsular contracture. Gel bleeding, involving minor silicone gel leakage through the intact implant shell, significantly promotes capsular contracture. Various pharmacological and surface treatment strategies have been explored to mitigate these issues.

**Objectives:**

This review assesses the effectiveness of different pharmacological interventions and BIs surface coatings on periprosthetic capsule development in murine models.

**Methods:**

A systematic review adhering to the PRISMA protocol was conducted. Databases searched included PubMed, Google Scholar, Cochrane Library, and LILACS using keywords: (Murine) AND (Breast) AND/OR (Implant), covering studies from 1977 to 2022. Experimental studies on miniature breast implants in murine models involving medications, surface treatments, or post-surgical therapies were included. Exclusions were studies without pharmacological agents, those testing bacterial contamination, radiotherapy, or involving different animal models or humans.

**Results:**

Twenty-nine articles were reviewed. Significant reductions in capsule thickness and inflammation were noted with certain pharmacological treatments. Corticosteroids and immunosuppressants were effective but raised concerns about wound healing and tumor recurrence. Leukotriene receptor antagonists (LTRA) showed promise in reducing capsule formation, especially in textured implants. Acellular dermal matrices (ADMs) enhanced tissue integration and reduced complications regardless of texture.

**Conclusions:**

Advancements have been made in therapies to influence capsular formation around silicone implants. However, clinical validation remains limited due to small sample sizes and short study periods. ADMs and LTRAs appear most promising, warranting further long-term clinical studies to fully understand their potential benefits in improving breast implant biocompatibility.

**No Level Assigned:**

This journal requires that authors assign a level of evidence to each submission to which Evidence-Based Medicine rankings are applicable. This excludes Review Articles, Book Reviews, and manuscripts that concern Basic Science, Animal Studies, Cadaver Studies, and Experimental Studies. For a full description of these Evidence-Based Medicine ratings, please refer to the Table of Contents or the online Instructions to Authors www.springer.com/00266.

## Introduction

Breast implants (BIs), one of the most common medical prostheses, have a history dating back to the second half of the nineteenth century. Before their implementation, several materials had been tried and tested with bemusing results, including wood, paraffin, sponges, and glass balls. It was only until the 1960s that modern silicone BIs were introduced [1]. Since then, BIs have advanced considerably through multiple generations, improving safety and tissue compatibility [2]. Current devices include a 6th generation which come into existence in the 2010s, featuring innovations such as biomimetic surfaces, enhanced shell resistance, and varying gel consistencies [3, 4].

Periprosthetic capsular development is a natural immune response occurring around any foreign body inserted inside the body, including BIs, significantly influencing implant safety and effectiveness [5]. Capsule characteristics, including thickness and tissue composition, can significantly impact the implant's function and the overall aesthetic outcome [6]. One of the most common long-term outcomes to BI placement is the onset of a periprosthetic capsule contracture around the implant, which is thought to occur due to an excessive fibrotic foreign body reaction to the implant [7]. Understanding biological and mechanical factors affecting capsular contracture onset is crucial for better therapeutic outcomes [8]. Several theories have been hypothesized, including the possible role of subclinical bacterial contamination and biofilm formation which may be associated with chronic inflammation and fibrotic immune response [9, 10]. Other theories attribute the increased likelihood of capsular contracture to external factors such as chest exposure to radiotherapy (i.e. post-mastectomy radiotherapy), which however only explains some of the cases [11].

Silicone bleeding through BIs shells has also been theorized to potentially cause capsular contracture, as this phenomenon has been reported to occur even without BI rupture [12, 13]. It involves minor leakage of silicone gel into surrounding tissues, which has been linked to higher risks of capsular contracture [14]. Anecdotal evidence reported in literature even mentioned the possibility of silicone-based internal mammary lymphadenopathy without any evidence of implant rupture [15]. Despite improved shell resistance of more modern BIs, gel bleeding persists and is a significant factor associated with higher Baker scores in capsules around smooth implants, rather than surface topography per se. Moyer et al. [16] found a dose-dependent correlation between gel bleeding and capsule stiffness (*p* < 0.05). Bakker et al. [17] also showed a significant correlation between silicone content in the capsule and capsular contracture severity, with higher silicone content in Baker grade IV capsules. The scientific community has focused on controlling the body’s reaction to BIs in order to affect or delay the onset of capsular contracture. Strategies include systemic and local therapies, and specialized implant surface covers, aimed at modulating the immune response to improve implant biocompatibility and overall success post-surgery.

The aim of this review was to evaluate existing knowledge in the literature regarding the impact of pharmacological interventions and different surface coatings on periprosthetic capsule formation in murine models. The review focused on studies using miniaturized BIs provided by manufacturers, analyzing various stages of capsule development, including inflammation, neoangiogenesis, collagen deposition, and fibrosis. Ultimately, the aim was to connect experimental findings with clinical applications to improve biomedical implant integration and functionality.

## Material and Methods

Our systematic review adhered to the Preferred Reporting Items for Systematic Reviews and Meta-Analyses (PRISMA) protocol. Researchers TM and GF searched PubMed, Google Scholar, Cochrane Library, and the Latin American and Caribbean Health Sciences Literature (LILACS) using keywords: (Murine) AND (Breast) AND/OR (Implant), without applying any time limits. We included experimental studies with murine models where only miniaturized breast implants or miniature replicas provided by companies where tested with external agents like medications, surface treatments, or post-surgical therapies. Exclusions criteria were studies without pharmacological agents, those focused on bacterial load inoculation to mimic subclinical or clinical infections, or radiotherapy, those involving different animal models or humans, and those where other types of materials were tested, different than miniature breast implants. Only English manuscripts were considered, excluding other languages. Reviews, brief communications, letters, and scholarly correspondence were used for reference but not included directly. Titles, abstracts, and full texts were independently reviewed by TM and GF, with disagreements resolved by consensus or third author MS.

**Fig. 1:**
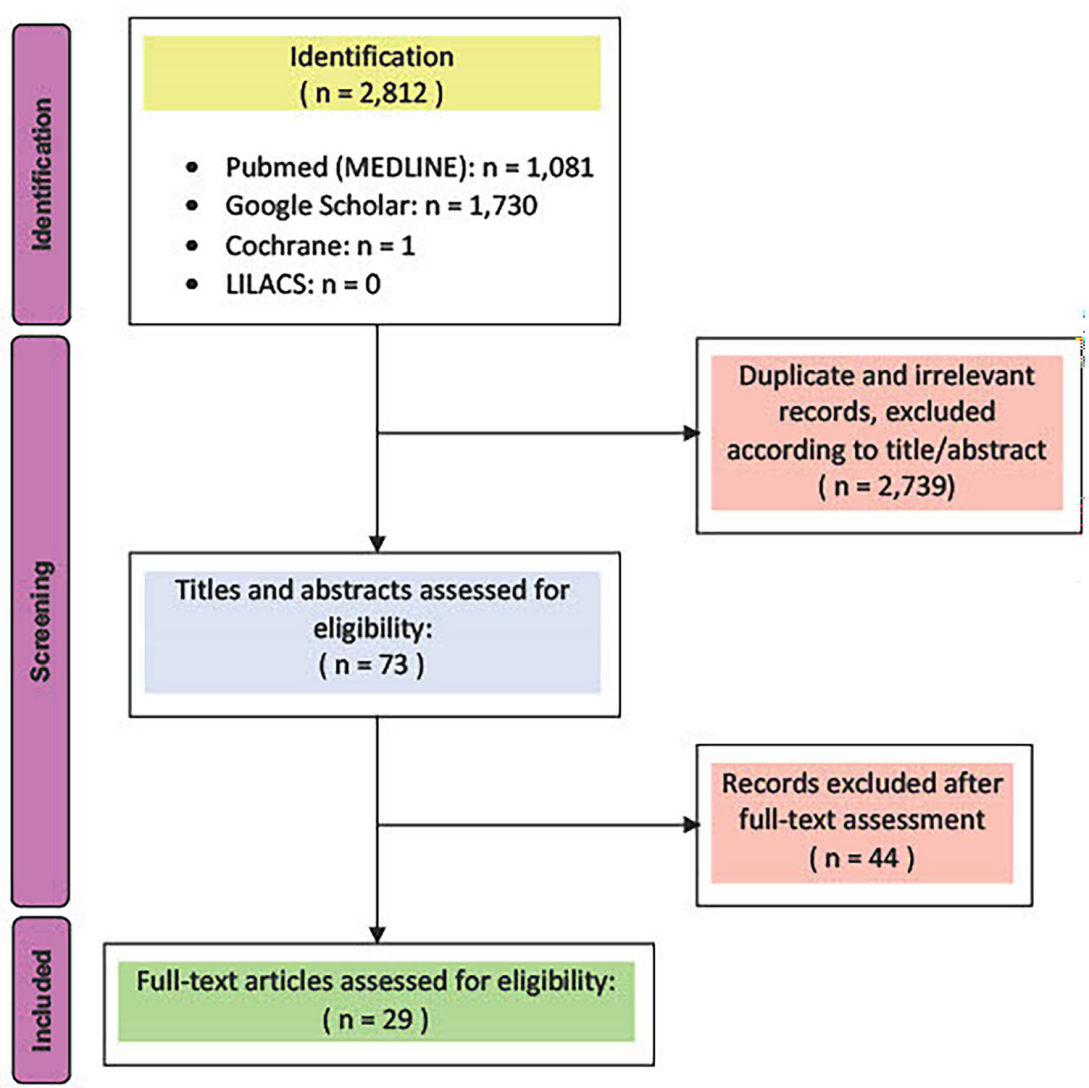
PRISMA-flowchart for manuscripts identification

A total of 2812 manuscripts were identified and after applying specified exclusion criteria, studies were narrowed down to 29 relevant full-text articles, ranging from 1977 to 2022. (Fig. 1). While reporting the results we followed the ISO 14607-2018 classification: smooth implants (< 10 µm), microtextured (10–50 µm), macrotextured and polyurethane (> 50 µm). Because of limited results on microtextured implants and many studies lacking implant surface characterization in µm, it was decided to include microtextured, macrotextured and polyurethane texturization in a single group, thus analyzing and discussing only smooth and textured.

## Results

### Smooth Implants

#### Anti-inflammatory Agents

##### Steroids

Moucharafieh et al. [18] found that triamcinolone diacetate (0.16 mg) alone or in conjunction with hematoma, did not significantly affect capsular thickness or intra-prosthetic pressure for smooth gel-filled BIs. Ksander et al. [19] compared control saline and methylprednisolone-filled implants with silicone rubber envelopes over 60 and 12 days. Cortisone implant capsules disintegrated upon removal, while control capsules were firm. Steroid levels dropped by 30–35% by 120 days. Rennekampff et al. [20] demonstrated similar results with smooth inflatable mini prothesis supplied by Heyer-Schulte Corp., Goleta, USA, filled with D-Penicillamine and Methylprednisolone at 40 days, with effects increasing with dosage. D-Penicillamine could be a corticosteroid substitute with fewer side effects.

##### Immunosuppressive Agents

Stark et al. [21] studied the impact of cyclosporine A on miniaturized smooth breast expanders (Cox-Uphoff, Santa Barbara, CA) in rats. The drug diffused through the silicone prosthesis's double-lumen outer shell. Histological analysis of the adjacent skin showed no changes, and capsules were statistically thinner in the cyclosporine-treated group after three months.

##### Leukotriene Receptor antagonists

Ozdemir et al. [22] found cromolyn sodium more effective than montelukast and zafirlukast in reducing inflammation and preventing capsular contracture around smooth implants at 90 days (Guangzhou Wanhe Plastic Materials Co. Ltd., Guangzhou, People’s Republic of China). However, Zafirlukast did not significantly affect smooth implants in terms of internal pressure or capsule formation, as highlighted by Bastos et al. [23, 24].

#### Anti-fibrotic Agents and Modulators

##### Statins

Chung et al. [25] found that Simvastatin (15 mg/kg/day) for 12 weeks significantly reduced capsular thickness and pro-fibrotic gene expression of TGF-β1 and CTGF around smooth-surfaced silicone BIs (SUN MEDICAL Co., Uiwang, Korea).

##### Omega-3 Fatty Acids

Lombardo et al. [26] reported that female C57BL/6 mice supplemented with Omega-3 oil daily for 12 weeks had significantly thinner capsules (205.09 mm) around smooth gel filled BIs (Mentor Corp., Santa Barbara, Calif.) compared to control group (361.63 mm), along with decreased TGF-β2 expression.

##### Other Fibrosis Modulators

Bae et al. [27] found that Periostin-deficient (PN-KO) mice, had thinner capsules around smooth silicone implants, fewer inflammatory cells, and lower fibrous tissue markers, indicating Periostin inhibition could reduce fibrous capsule formation. Gancedo's [28] research showed that Pirfenidone (200 mg/kg daily for 8 weeks) significantly lessened capsule thickness, reduces fibroblast growth and inflammation, and decreased collagen levels around both smooth and textured silicone implants, showcasing its anti-fibrotic properties without statistical difference between both surfaces.

#### Barrier Solutions and Surface Coatings

Yang's [29] study found combined GuardiX® and Montelukast therapy significantly decreased inflammatory and fibrotic markers around smooth silicone implants at 8 weeks. Bergman et al. [30] found no significant differences in capsular thickness or blood vessel infiltration between saline-filled titanium-coated silicone implants and saline-filled silicone implants at 36 weeks.

#### Stem Cell-Based Treatments

Kim et al. [31] studied the effect of human embryonic stem cell-derived endothelial precursor cell conditioned medium (hESC-EPC CM) on capsule formation around smooth silicone implants. After two months, treated implants had thinner capsules and improved angiogenesis without adverse effects.

#### Physical and Mechanical Therapies

Mendes et al. [32] applied ultrasound at 3 MHz frequency around smooth silicone implants (Silimed, Rio de Janeiro, RJ) and, three times weekly for 90 days. At 180 days, ultrasound-treated group had thicker capsules, more cells, better blood vessel formation, and less organized collagen fibers. Cardenas-Camarena et al. [33] used electrostimulation on 20 smooth and 20 textured Nagor gel implants, finding that currents above 300 mV, especially 600 mV, significantly reduced periprosthetic capsule formation regardless the type of surface texture..

All the results pertaining to interventions and their outcomes on smooth implants are comprehensively summarized in Table 1.

**Table 1:** Interventions and outcomes for smooth implants

Intervention type	Mechanism of action	Key outcomes	Clinical relevance
Corticosteroids (Triamcinolone, Methylprednisolone)	Anti-inflammatory; reduces fibrosis	No statistical significance for triamcinolone diacetate Methylprednisolone disintegrated peri-prothetic casule	Promising; concerns about wound healing
Immunosuppressive agents (Cyclosporine A)	Suppresses immune response via calcineurin	Statistically thinner capsules	Effective; Potential effects on tumor recurrence
Leukotriene Receptor Antagonists (Montelukast, Zafirlukast)	Blocks leukotriene-induced inflammatory pathways	Reduced capsule thickness and inflammation	Promising for smooth implants; warrants further study
Statins (Simvastatin)	Anti-fibrotic; inhibits TGF-β1	Decreased capsule thickness, reduced pro-fibrotic gene expression	Strong anti-fibrotic properties; minimal side effects
Omega-3 Fatty Acids	Anti-inflammatory; reduces TGF-β expression	Significantly thinner capsules, reduced inflammatory markers	Potential adjunctive therapy; requires further validation
Periostin Inhibition (PN-KO Mice)	Reduces fibrotic tissue markers	Thinner capsules, fewer inflammatory cells and fibrous tissue markerspi	Promising genetic model; limited translational application
Pirfenidone	Anti-fibrotic; reduces fibroblast growth and collagen deposition	Significantly thinner capsules, reduced inflammation and fibroblasts in both smooth and textured surfaces	Effective across implant surfaces; warrants further study
Barrier Solutions (GuardiX®)	Creates barrier reducing inflammatory response	Reduced inflammatory markers, thinner capsules	Combination therapy; promising for clinical applications
Stem Cell Therapy (hESC-EPC CM)	Promotes angiogenesis and reduces fibrosis	Thinner capsules, improved vascularization	Promising; requires validation for safety and efficacy
Physical Therapy (Ultrasound)	Mechanical disruption of capsule formation	Increased capsule thickness and angiogenesis, disorganized collagen fibers	Potentially counterproductive; limited clinical application

### Textured Implants

#### Anti-inflammatory Agents

##### Steroids

Moreira et al. [34] found that a single dose of liposome-derived prednisolone, resulted in thinner capsules, lower collagen density, and fewer myofibroblasts around silicone gel-filled textured implants at 90 days (Silimed, Brasil, Sao Paulo, Brazil).

##### Leukotriene Receptor Antagonists

Multiple studies confirmed Zafirlukast’s effectiveness in reducing capsule thickness, vascularization, and collagen density around textured implants. Bastos et al.^25^ reported fewer vessels and thinner capsules around textured implants at 90 days with daily intraperitoneal injections of Zafirlukast 5 mg/kg. Moreira et al. [35] noted reductions in capsule thickness, collagen density, and myofibroblast counts from a single dose administered during surgery around textured, shell-shaped silicone implant (Silimed, Brasil, Sao Paulo, Brazil) with effects at both 35 and 90 days.

#### Anti-fibrotic Agents and Modulators

Zimman et al. [36] found Enalapril reduced TGF-β1 levels and inflammatory infiltrate at three months, with better results in textured devices (Nagor Ltd., England) when compared with smooth surfaces (Nagor Ltd., England). Zeplin et al. [37] demonstrated that textured miniature silicone implants (Nagor Ltd., London, England) coated with Halofuginone lactate, had significantly thinner capsules with fewer inflammatory cells and fibroblasts. Additionally, they found that Phosphorylcholine-coated (PC) textured silicone implants significantly reduced fibrous capsule formation and inflammation at three months, due to their hydrophilic properties [38]. Luan et al. [39] discovered that a 40 mg/mL concentration of paclitaxel significantly reduced capsule thickness around textured miniature silicone breast implants (Shanghai Winner Plastic Surgery Products Co., Ltd. China) compared to the control group. Concentrations below 20 mg/mL or above 80 mg/mL were less effective or caused adverse effects, emphasizing the importance of proper dosing. Di Lido's [40] study on histone deacetylase (HDAC) inhibitors showed that after 30 days, mice treated with MC2625 and MC2780 had significantly thinner fibrous capsules around textured silicone implants (Allergan, Inc., Irvine, CA), with increased IL-10 levels and reduced IL-1β and IL-6.

#### Barrier Solutions and Surface Coatings

Berger et al. [41] found that microtextured silicone implants LifeSil® (Curitiba, PR, Brazil) with Parietex® mesh, developed thicker capsules, but collagen levels were consistent with implants without mesh. Schmitz et al. [42] showed that Acellular Dermal Matrix (ADM)-covered textured silicone testicular implants (Neuticles Ultra plus XX small, Neuticles CTI Corporation, Oak Grove, MO, USA) significantly reduced inflammation and created a thinner myofibroblast layer at 12 weeks. Ludolph et al. [43] found that same implants wrapped in acellular porcine dermis had a significantly thinner collagenous layer, fewer myofibroblasts, and reduced TGF-β1 expression at 12 weeks, with further improvements at 52 weeks, including increased angiogenesis.

Bernardini et al. [44] tested smooth, textured, and polyurethane-covered mini implants (SILIMED-TM, Company, São Paulo, Brazil), covered with two types of bovine acellular pericardial meshes (APMs), BioRipar (ASSUT-EUROPE, Rome, Italy), and Tutomesh (Tutogen Medical Gmbh, Neunkirchen am Brand, Germany). Implant texture did not significantly affect tissue remodeling when used with APMs, but BioRipar caused less inflammation and promoted better blood vessel growth than Tutomesh.

#### Physicial and Mechanical Therapies

Yarar et al. [45] evaluated the effect of hyperbaric oxygen (HBO) on capsule formation around textured silicone miniaturized BIs, showing that HBO therapy significantly reduced capsule thickness, fibroblasts, neutrophils, and macrophages at 60 days, regardless the implant was placed over or under the muscle.

Fischer et al. [46] studied extracorporeal shock wave therapy (ESWT) on capsule formation around textured silicone gel implants (Polytech Health and Aesthetic, Dieburg, Germany) finding that multiple ESWT sessions over 14 days, significantly reduced capsule thickness at 100 days without affecting myofibroblast levels and vessel density.

All the results pertaining to interventions and their outcomes on textured implants are comprehensively summarized in Table 2.

**Table 2:** Interventions and outcomes for textured implants

Intervention type	Mechanism of action	Key outcomes	Clinical relevance
Corticosteroids (Prednisolone)	Anti-inflammatory; reduces fibrosis	Thinner capsules, lower collagen density, fewer myofibroblasts	Promising; concerns about wound healing
Leukotriene Receptor Antagonists (Zafirlukast)	Blocks leukotriene-induced inflammatory pathways	Reduced capsule thickness, myofibroblast counts, and vascularization	High potential; further studies needed for long-term efficacy
Angiotensin-converting enzyme (ACE)-Enalapril	Inhibits fibrosis via TGF-β suppression	Reduced TGF-β1 levels significantly Decreased inflammatory cell infiltrate	Promising anti-fibrotic intervention. More effective for textured implants
Anti-fibrotic Agents (Halofuginone Lactate)	Inhibits fibrosis via TGF-β pathway	Reduced capsule thickness significantly Decreased fibroblast and collagen production	Promising for textured implants; requires safety studies
Paclitaxel	Mitotic inhibitor; Stabilizes microtubules, blocking fibroblast proliferation and reducing fibrosis	Significantly thinner capsules; dose-dependent effects	Requires precise dosing; potential side effects
Histone Deacetylase (HDAC) Inhibitors	Modulates epigenetic pathways to reduce inflammation and fibrosis progression	Significantly thinner capsules; Increased IL-10, Decreased IL-1β and IL-6	Promising anti-inflammatory agent; requires further study
Phosphorylcholine Coating	Mimics membranes, prevents adhesion, reduces inflammation, and fibrosis	Significantly thinner capsules, and fewer inflammatory cells	Effective hydrophilic coating
Parietex® mesh	Provides scaffold for cellular ingrowth	Thicker capsules Consistent collagen levels with implants without mesh	Promising; Requires further study
Acellular Dermal Matrices (ADM)	Enhances tissue integration, reduces immune response	Reduced myofibroblast activity, inflammation and induced thinner collagen layers	Strong potential for reducing complications; limited long-term data
Hyperbaric Oxygen Therapy	Enhances oxygenation to reduce inflammation and fibrosis	Significantly thinner capsules, fewer neutrophils, macrophages and fibroblasts	Promising non-invasive therapy
Extracorporeal Shock Wave Therapy	Disrupts capsule formation through mechanical stimulation	Significantly reduced capsule thickness, no effect on vessel density	Promising adjunct therapy; requires further validation

## Discussion

Surface characteristics and gel bleeding, which inevitably occurs through the implant’s shell, both condition capsular formation. Efforts to influence host inflammatory responses and capsular development through various therapies have been made, but clinical validation still remains elusive. Additionally, this phenomenon is insufficiently investigated. Thicker shells may reduce silicone migration, while saline-filled implants pose fewer risks upon leakage, though they bring concerns of aesthetic outcomes and higher deflation risk [47].

Steroids shows to reduce thickness myofibroblast and collagen density when used around textured implants (prednisolone), and also when using Methylprednisolone in combination with D-Penicillamine around smooth implants, being the latter a better alternative due to fewer wound healing issues.

Immunosuppressive agents (cyclosporine A) were evaluated only on miniaturized smooth breast expanders, showing statistically thinner capsules but with major concern for local immunosuppressive effects on local tumor onset and recurrence due to their diffusion through the shell.

Leukotriene Receptor antagonists demonstrated to reduce inflammation and prevent capsular contracture around smooth implants, however Zafirlukast did not significantly affect internal pressure or capsule formation. On contrary Zafirlukast confirmed its effectiveness in reducing capsule thickness, myofibroblast counts, vascularization, and collagen density around textured implants. This varying efficacy, with cromolyn sodium more effective on smooth implants, while zafirlukast on textured implants, can likely be due to the stronger inflammatory response elicited by textured implants, enhancing the clinical impact of anti-inflammatory medications. Unfortunately most of this studies are limited by a small sample size, lack of control group, and short duration.

The use of anti-fibrotic agents significantly reduced capsule thickness in both smooth and textured implants. Simvastatin, Omega-3 fatty acids, and Periostin-deficient mice showed promising results in smooth implants. Halofuginone lactate, paclitaxel, and HDAC inhibitors were more effective in textured implants. Pirfenidone reduced capsule thickness in both surfaces without significant differences. Enalapril was more effective in textured devices, reducing TGF-β1 levels and inflammatory infiltrate better than in smooth surfaces.

Barrier solutions and surface coatings effectively reduced capsular formation. Combined GuardiX® and Montelukast therapy decreased inflammatory and fibrotic markers in smooth implants. Acellular dermal matrices (ADMs) modulated immune responses and influenced capsular development regardless of implant texture. Cottler el al. [48] showed that using ADM to deliver IL-4, significantly enhanced angiogenesis, improving dermal matrix integration and clinical outcomes. IL-4 is known to promote M2 macrophage polarization, which has anti-inflammatory properties. On a clinical level, Samaha et al. [49] showed that implant-based breast reconstruction with ADM, especially prepectoral, led to fewer need for more surgeries and less incidence of capsular contracture, but higher risk of infection and wound complication, even though the results are limited to only one institution and are focusing solely on short-term complications.

Stem cell treatments, like human embryonic stem cell-derived endothelial precursor cell conditioned medium (hESC-EPC CM), tested only around smooth implants show promise in thinning capsules and improving angiogenesis, but more research is needed to confirm efficacy and safety.

Physical therapies showed mixed results. Ultrasound at 3 MHz thickened capsules around smooth implants, while electrostimulation over 600 mV significantly reduced capsule formation for both surfaces [35]. Hyperbaric oxygen (HBO) and extracorporeal shock wave therapy (ESWT) significantly reduced capsule thickness and inflammation in textured implants.

Those results can possibly be explained due to a bio-tribological effect which is supported by many other authors who found that higher surface texturization can significantly modify capsule characteristics and development due to micromotions that can generate shear stress which sustains apoptosis, chronic inflammation, synovial metaplasia and macrophage polarization in M1 state which is pro-inflammatory [50–53]. In this pro-inflammatory environment, DNA damage may appear and suppresses immune-surveillance against cancer cell, potentially promoting breast implant associated malignancies.^52^

Notably, Rifampicine was not found in any eligible studies to be included in the manuscript, despite its widespread use in addressing implant related complications, and not only in breast surgery, but also in other specialties where implantable devices are used [54]. However, it showed positive results in rabbit models, reducing the capsular contracture rate around saline-filled implants by potentially combating the subclinical bacterial contamination, one of the theories of capsular contracture [55].

Many studies face limitations such as small sample sizes, short observation periods, and potential systemic reactions or alterations in the breast environment, which may promote tumor recurrence, implant extrusion, or other complications. Lack of standardized protocols and the inconsistent reporting of implant surface roughness (often not expressed in µm or according to ISO 2018 or the new ISO 2024 classification) further complicate the comparison and reliability of those findings. Moreover, none of the studies reviewed adhered to the ARRIVE guidelines or the Gold Standard Publication Checklist, highlighting the need for greater standardization in animal research. Methodological variability, including differences in surgical techniques, follow-up durations, and animal strains, remains a limitation, however, we ensured consistency by including studies using miniaturized implants produced by reputable manufacturers for scientific purposes, all placed in the same surgical plane. Although murine models rarely fully induce clinical capsular contracture spontaneously, they remain established models for assessing pharmacological interventions at the cellular level, providing valuable insights into the early stages of capsule formation and bridging the gap between preclinical data and human studies.

Several studies included in this review received miniature breast implants, medications and other necessary materials as donations from various companies. Ensuring transparent disclosure of industry relationships and financial ties in academic papers is vital for preserving research credibility and integrity. This requirement supports the ongoing dialogue in the scientific community about addressing conflicts of interest (COIs) to maintain unbiased and reliable scientific results for readers and fellow researchers [56]. Corporate-sponsored research often shows low complication rates, fast recoveries, high patient satisfaction, and high conversion rates, which compromises the integrity of honest reporting and diminishes the emphasis on evidence-based medicine in scientific literature [57].

## Conclusion

Overall, researchers have made significant advancements in exploring therapies to influence capsular formation around silicone implants. Still, most findings lack solid clinical backing due to small sample sizes and short study periods thus having limited understanding of potential side effects. Further most of those results active on capsule formation are biased by the fact that have been used only in combination to one implant surface, known to be highly responsible for foreign body reaction, thus limiting the understanding of the efficacy of the agent. Similarly the different degree of capsule formation between different surfaces can influence the final efficacy of the agent in improving their characteristics. Finally the use of ADM meshes and leukotriene receptor antagonists seem to have the most encouraging results so far, with limited side effects, making further comprehensive long-term clinical studies important in understanding their potential benefits in improving breast implants biocompatibility.
